# B7-H3 targeted CAR-T cells show highly efficient anti-tumor function against osteosarcoma both *in vitro* and *in vivo*

**DOI:** 10.1186/s12885-022-10229-8

**Published:** 2022-11-02

**Authors:** Qian Zhang, Zhiqiang Zhang, Guodi Liu, Dehua Li, Zhangjie Gu, Linsong Zhang, Yingjiao Pan, Xingbing Cui, Lu Wang, Guoping Liu, Xiaoli Tian, Ziming Zhang

**Affiliations:** 1 Shanghai Yihao Biological Technology Co., Ltd, Shanghai, 200231 China; 2 Department of Pediatric Orthopedics, National Children’s Medical Center & Children’s Hospital of Fudan University, Shanghai, 201102 China; 3 State Key Laboratory of Bioreactor Engineering, East China University of Science and Technology, Shanghai, 200237 China; 4 Department of General Surgery, Changhai Hospital, Shanghai, 200433 China; 5 Shanghai Beautiful Life Medical Technology Co., Ltd., Shanghai, 200231 China; 6 Xinhua Hospital Affiliated to Shanghai Jiaotong University School of Medicine, 1665 Kongjiang Road, Shanghai, 200092 China; 7 Department of Orthopaedics, Shanghai Children’s Hospital, School of Medicine, Shanghai Jiao Tong University, Shanghai, 200062 China

**Keywords:** Osteosarcoma, B7-H3, Chimeric antigen receptor T, Patient-derived xenografts

## Abstract

**Background:**

Osteosarcoma (OS) mainly happens in children and youths. Surgery, radiotherapy and chemotherapy are the common therapies for osteosarcoma treatment but all their anti-tumor effects are limited. In recent years, a new cellular therapy, CAR-T, a cellular immunotherapy with genetically engineered T cells bearing chimeric antigen receptor targeting specific tumor-associated antigen, has been proved to be an effective therapy against acute lymphoblastic leukemia. Thus, CAR-T is a potentially effective therapy for osteosarcoma treatment.

**Methods:**

A CAR gene targeting B7-H3 antigen was constructed into lentiviral vector through molecular biology techniques. Then, the CAR gene was transferred to T cells through lentiviral delivery system, and the CAR-T cells were largely expanded using *in vitro* culture technology. The *in vitro* anti-tumor effect of CAR-T cells was evaluated through Real Time Cell Analysis system (RTCA) and ELISA assay. The *in vivo* anti-tumor capabilities of CAR-T cells were evaluated using the patient-derived xenografts (PDX) model of osteosarcoma.

**Results:**

The third-generation CAR-T cells we constructed could target the B7-H3 antigen, and the phenotype of CAR-T cells was consistent with normal T cells; The CAR-T cells showed superior antitumor effects both *in vitro* and *in vivo*.

**Conclusion:**

Our study showed that B7-H3 targeted CAR-T cells had high anti-tumor efficacy against osteosarcoma both *in vitro* and *in vivo*, which proved that B7-H3 targeted CAR-T therapy is potentially effective for osteosarcoma treatment.

**Supplementary Information:**

The online version contains supplementary material available at 10.1186/s12885-022-10229-8.

## Background

Osteosarcoma(OS)is one of the most common malignant bone tumors, which occurs most frequently in children and adolescents. At present, the treatment of osteosarcoma focuses on the combination of surgery, radiotherapy and chemotherapy [1, 2]. However, the effects of the current treatments were not significantly improved, and some patients soon developed recurrence or lung metastasis after using the current treatments [3, 4]. Since the anti-tumor immune mechanism of osteosarcoma is not fully defined, it is particularly important to develop more efficient immunotherapy methods in the treatment of osteosarcoma for prolonging the survival cycle of patients and reducing the toxic and side effects and metastasis rate.

In recent years, chimeric antigen receptor-T(CAR-T) cell therapy has emerged as one of the most promising immunotherapy methods. The CAR-T cells can effectively kill tumor cells through specifically recognizing and binding antigens on tumor cell membranes [5]. CAR-T cell immunotherapy has shown excellent efficacy in the treatment of hematologic tumors [6]. In clinical trials, since CD19 targeted CAR-T cells showed excellent efficacy in the treatments of patients with hematologic malignancies, the FDA has approved CD19 CAR-T cells as new therapies for non-Hodgkin's lymphoma and chemotherapy-refractory/relapsed acute lymphoblastic leukemia treatments [7]. However, In the treatment of solid tumors, the efficacy of CAR-T cell immunotherapy is not significant when compared with the treatment of blood tumors. The lack of efficacy in CAR-T therapy treating solid tumors is composed of multiple factors including the immune escape mechanism of targeted tumor cells, the reduction of CAR-T cells transport to tumor cells, the tumor cell microenvironment, and many other factors [8]. For improving the effectiveness of CAR-T cell immunotherapy in solid tumor treatment, we must focus on the following criteria: CAR-T cells should target epitopes that are selectively expressed on the surface of tumor cells; the target must be widely expressed on tumor cell metastasis. At present, CAR-T therapies that specifically targeting different kinds of solid tumor associated antigens have been studied in the treatment of osteosarcoma, and the tumor associated antigens mentioned above included human epidermal growth factor receptor 2 (HER2) [9, 10], activated leukocyte adhesion molecules (ALCAM, CD166) [11], GD2 [12], and interleukin IL-11 receptor alpha (IL-11Rα) [13]. However, for the purpose of quickly bringing the CAR-T therapies for the treatment of osteosarcoma to market, developing new excellent solid tumor associated antigens for CAR-T therapies is still important.

B7-H3 (CD276) is one type I transmembrane glycoprotein, which belongs to the B7 costimulatory molecule family. In humans, there are two subtypes of B7-H3 protein, namely 2IGB7-H3 and 4IGB7-H3 [14, 15]. B7-H3 has immunosuppressive functions, which can reduce T cell-released type I interferon (IFN) and cytotoxic activity of natural killer cells [16]. Previous studies indicated that the expression level of B7-H3 protein was high in a variety of human cancers, such as prostate cancer, breast cancer, neuroblastoma, glioma, colorectal cancer, pancreatic cancer, and other solid cancers, but low or no in normal human tissues [17–21]. It is reported that the expression level of B7-H3 is closely related to tumor metastasis, tumor immune escape, prognosis, and clinical outcome [22, 23]. All above show that B7-H3 is a potential target for tumor immunotherapy. There are many approaches to utilize B7-H3 for tumor treatment in preclinical or clinical trials, such as bi-specific antibodies, antibody–drug conjugation therapy, and drugs targeting B7-H3 through antibody-dependent cell-mediated cytotoxicity, CAR-T cell therapy [24]. Among the above approaches, B7-H3 CAR-T cell therapy has been used for treating a variety of solid tumors including neuroblastoma, glioblastoma, atypical teratoma/rhabdomyoma, Ewing's sarcoma, which indicated that B7-H3 was an excellent target for CAR-T cell therapy [20, 25–27].

Based on all above, in our study, with the goal of obtaining one excellent CAR-T product for osteosarcoma treatment, we constructed B7-H3 targeted CAR-T cells, then we evaluated anti-tumor effects of B7-H3 targeted CAR-T cells both *in vitro* and *in vivo* using the patient-derived xenografts (PDX) model of osteosarcoma. Consequently, B7-H3 targeted CAR-T cells we developed showed highly anti-tumor efficacy against osteosarcoma and had the enormous potential to be an excellent CAR-T cell product in the future.

## Materials and methods

### Construction of the B7-H3 targeted CAR vector

The third generation B7-H3 CAR contains the B7-H3 specific target single chain antibody fragment (scFv) with a CD8 leading sequence, a CD8 hinge and transmembrane sequence, as well as the intracellular signaling domain of 4-1BB, CD28 and CD3ζ in tandem. The sequences except scFv are consistent with those used in our previous report [28]. The full-length nucleotide sequence was synthesized by biotechnology company (Sangon Biotech, Shanghai, China), and were subsequently inserted in lentiviral CAR expression vector through two specific restriction enzyme sites.

### Production of lentivirus and T cell transduction

HEK293T cells were transiently transfected with B7-H3 CAR lentivirus vector, package plasmids PLP1-K2, PLP2-K2 and PLP-VSVG-K2 at a ratio of 23.1:16.5:16.5:9.9. Polyethyleneimine (Polyscience, Warrington, PA, USA) was used as DNA transfection reagent. Collecting the cell supernatant at 48 h and 72 h after transfection. After centrifugation at 4000 rpm for 5 min to remove cell debris, filtered the supernatant with a 0.45 μm filter, added PEG8000 (Sigma, Merk, Shanghai, China) to the supernatant and placed it at 4 °C overnight. Then, the supernatant was centrifuged at 4000 g for 30 min for concentration. Lentivirus particles were resuspended using PBS and stored at -80 °C after viral titer determination.

With the approval of the Ethics Committee of Xinhua Hospital, fresh blood from healthy donors was extracted with the informed consent signed by the informed person. Human peripheral blood mononuclear cells (PBMCs) were isolated with a density gradient using the Ficoll (GE) kit. T cells were activated by magnetic beads with anti-human CD3 Ab (100 ng/mL; T&L Biotechnology) and anti-human CD28 Ab (100 ng/mL; T&L Biotechnology). Activated T cells were proliferated in X-VIVO 15 medium (Lonza) containing recombinant human IL-2 (30 ng/mL; Novoprotein) and 1% penicillin–streptomycin(Gibco, Life Technologies, Shanghai, China). One day after activation, the activated T cells were transduced by lentiviral particles under a determined infection complex number, and the medium was changed every 2–3 days. Then the cells were continuously cultured for 12–14 days, and the cells were collected for *in vivo* and *in vitro* experiments. Non-transduced T cells were prepared for control experiments. Each *in vivo* and *in vitro* experiment was repeated with T cells from different healthy donors.

### Cell lines and culture conditions

HEK293T cells, human osteosarcoma cell lines including HOS, U-2 OS, SW1353 and Saos-2, and human leukemic T lymphocytes Jurkat were obtained from the Chinese Academy of Cell Bank Sciences (Shanghai, China). HEK293T cells was cultured in Dulbecco's modified DMEM medium (Gibco, Grand Island, NY, USA). HOS, U-2 OS, SW1353 and Saos-2 were maintained in MEM medium (Gibco, Grand Island, NY, USA). Jurkat cells were maintained in 1640 medium (Gibco, Grand Island, NY, USA). All cell lines were supplemented with 10% heat-inactivated fetal bovine serum (Gibco, Life Technologies, Shanghai, China) and 1% penicillin–streptomycin (Gibco, Life Technologies, Shanghai, China). All cell lines were cultured in an incubator at 37 °C with 5% CO_2_.

### Immunohistochemistry (IHC)

Samples of human osteosarcoma were obtained from Xinhua Hospital. All the tissue samples were used for scientific research with the consent of people familiar with the matter. Commercial antibodies of B7-H3 were used to stain the tissue samples, and all the steps of IHC staining experiment were carried out were as follows: In short, all tissue samples were fixed with 4% paraformaldehyde, dehydrated and transparent, embedded in paraffin, sliced ​​with 4 μm thickness, deparaffinized and rehydrated. B7-H3 specific antibody (Sino Biological Inc.) was used for immunostaining at 4℃ overnight. Then the horseradish-peroxidase (HRP) labeled goat-rabbit immunoglobulin secondary antibody (ZSGB-BIO, Beijing, China) was stained, and DAB was added for coloration. Finally, Olympus BX53 microscope (Japan) was used for microscope observation.

### Flow cytometry and antibodies

Flow cytometry cell staining was performed using PBS solution supplemented with 2% FBS and 2% EDTA at 4 °C. Recombinant B7-H3 FITC conjugated antibody (Sino Biological Inc., Beijing, China) was used to detect the expression of B7-H3 protein on cancer cells by flow cytometry. Recombinant human B7-H3 protein (Novoprotein, Shanghai, China) was used to detect the expression of B7-H3 CAR on CAR-T cells, and then stained with allophycocyanin (APC) anti-His Tag (BioLegend, CA, USA).

The antibodies using in immunophenotypes of T cells by flow cytometry were as follows: APC-Cy 7 mouse anti-human CD3 ( BD, NJ, USA), APC mouse anti-human CD4 (BD, NJ, USA), PE mouse anti-human CD8 (BD, NJ, USA), Brilliant Violet 421 anti-human CD197 (Biolegend, CA, USA) FITC anti-human CD45RA (Biolegend, CA, US), PE-Cy7 anti-human CD45RO (Biolegend, CA, USA), Brilliant Violet 421 anti-human CD197(CCR7) (Biolegend, CA, USA), Brilliant Violet 510 anti-human CD62L (Biolegend, CA, USA), Brilliant Violet 605 anti-human CD95(Fas) (Biolegend, CA, USA), PE anti-human CD223(LAG-3) (Biolegend, CA, USA), Brilliant Violet 421 anti-human CD366(Tim-3) (Biolegend, CA, USA), and APC anti-human CD279(PD-1) (Biolegend, CA, USA). Select the appropriate test channel according to the manufacturer's product instructions for flow cytometry. The cells were then obtained using flow cytometer NovoCyte D3000 (ACEA, Ashland, OR, USA) and the data were analyzed using NovaExpr software (ACEA, Ashland, OR, USA).

The the blood of the mouse model was collected and treated with RBC Lysis Solution (QIAGEN, Shanghai, China), then the blood was detected by flow cytrometry accordding to the operation above. Mouse tumor tissue was digested using collagenase IV (Sigma, Merk, Shanghai, China) and trypsin (Gibco, Grand Island, NY, USA) for flow cytrometry.

### Cytotoxicity assays

Tumor cells were inoculated at a concentration of 2 × 10^4^ cells per well in a specific 96-well E-plate (Acea Biosciences, Menlo Park, CA, USA). After 20 h of culture, non-transduced-T cells (control group, named NC-T) and B7-H3 targeted CAR-T cells (experimental group, named B7-H3 CAR-T) were added to the 96-well E-plate in different proportions (E:T of 2.5:1, or 1:1). The group without T cells was tumor-only group (named Tumor Only). The 96-well E-plate plate was then incubated in an incubator at 37 °C with 5% CO_2_. RTCA software (Xcelligence RTCA SP, ACEA, Los Angeles, CA, USA) was used to detect the viability of target cells in real time.

### Analysis of cytokine secretion

Tumor cells were co-cultured with NC-T cells and B7-H3 CAR-T cells in a 96-well plate without adding any exogenous cytokines. Then, the supernatant was collected after 24 h. The cytokines (IFN-γ and TNF-α) were determined using specific enzyme-linked immunosorbent (ELISA) kits (eBioscience, Grand Island, NY, USA).

### Tumor models and treatment

The 6–8 weeks old NODPrkdcem26IL2rgem26/Nju (NCG) mice used in the experiment were purchased from NBRI (Nanjing, China). The mice were raised under conditions without specific pathogens, and all procedures were carried out under the guidance of the Shanghai Beautiful Life Animal Center and the Institutional Animal Welfare Committee.

For constructing the PDX models, surgical osteosarcoma tumor samples were obtained from the Xinhua Hospital (Shanghai, China) with informed consent from the patients. The tumor samples were cut into fragments with a volume of about 4-5mm^3^ and subcutaneously transplanted into NCG mice. When the transplanted tumor volume reached 50–100 mm^3^, the mice were divided into four groups with 5 mice per group. The experiment must be stopped immediately and euthanize the mice when any of the following occurs: The size of the tumor exceeds 2000mm^3^, the weight of the mouse decrease by more than 25%, the mice cannot eat for more than 48 h, the tumor ulcerates or causes significant pain, and the tumor affects the normal movement and behavior of the mice. Tumor size was measured twice per week with a caliper, and the tumor volume was calculated by the following equation: volume = (length × width^2^)/2, where length represented the longest dimension.

### Statistical analysis

Statistical analysis and graph generation were performed using GraphPad Prism Software v.7.0 (La Jolla, CA). All data were expressed as mean ± SD. All experiments in this study were repeated at least 3 times. The student t-test was used to compare the two groups, while for multi-group comparisons, one-way or two-way ANOVA was used to determine statistically significant differences between the samples. The *p* value < 0.05 was considered to be significant, and the significance level was shown in the figure as *p < 0.05, **p < 0.01, and ***p < 0.001.

## Results

### Expression of B7-H3 target in osteosarcoma samples and tumor cell lines

**Fig. 1:**
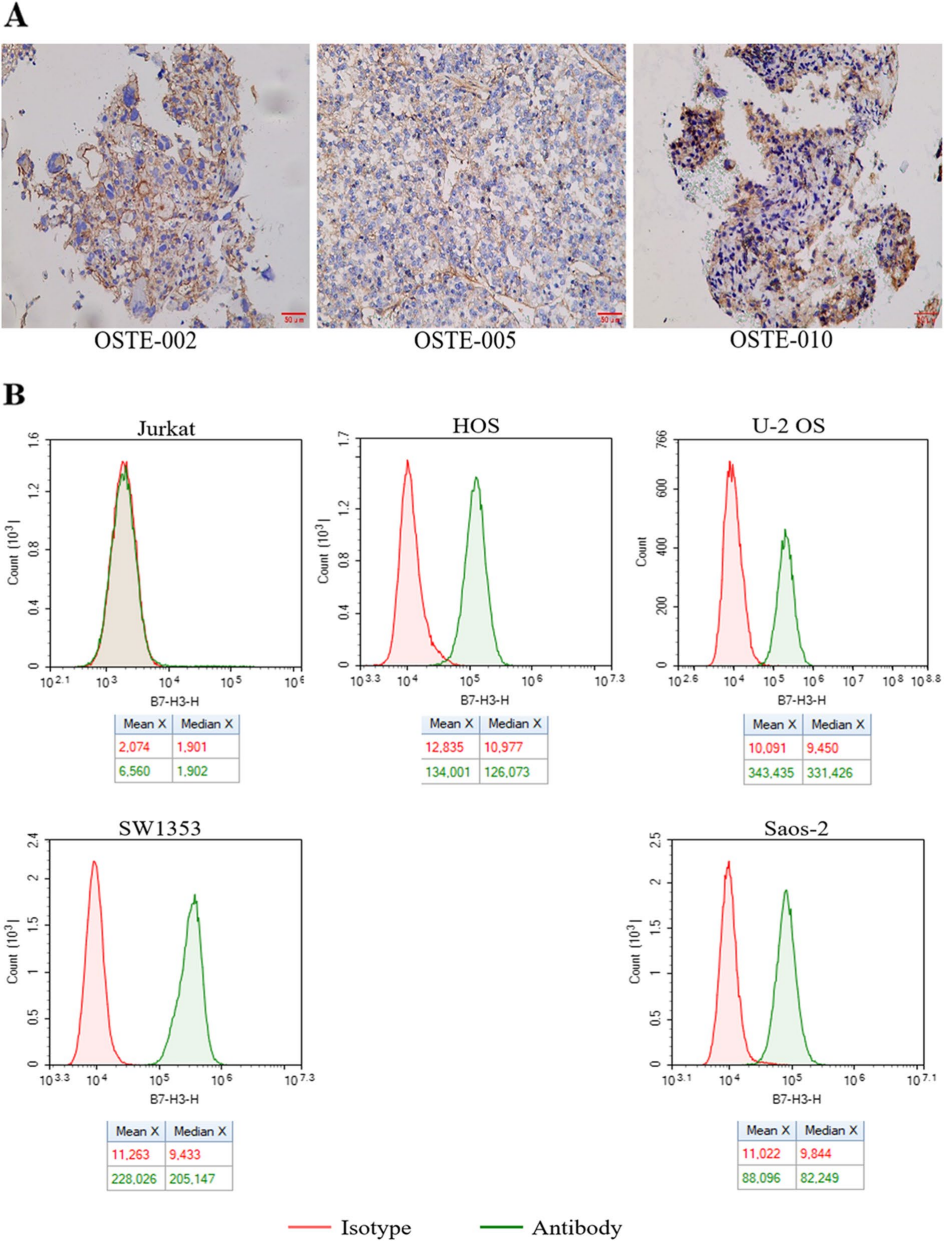
Expression of B7-H3 target in osteosarcoma samples and tumor cell lines. **A** B7-H3 expression was detected in OS tissues. OS Osteosarcoma. scale bar = 50 μm. **B** The surface expression of B7-H3 receptor level on tumor cell lines (Jurkat, HOS, U-2 OS, SW1353, Saos-2) detected by flow cytometry

To determine whether B7-H3 could be a therapeutic target for osteosarcoma, we performed immunohistochemical analysis of B7-H3 protein on 60 pathological sections of osteosarcoma from clinical hospitals. The IHC staining results were assigned a mean score based on both the intensity of staining and the percentage of positive cells. The IHC intensity was scored as follows: no staining (0 point), minimal staining (light yellow) (1 point), moderate staining (yellow brown) (2 points), and strong staining (brown) (3 points), respectively. The percentage of positive cells was determined and divided into five groups: < 5% positive cells (0 point), < 5–25% positive cells (1 point), 26–50% positive cells (2 points), 51–75% (3 points) and 76–100% positive cells (4 points). The IHC was scored using a composite scoring system: scores were calculated by multiplying the intensity with the percentage of positive cells having this intensity. 0, 1–4, 5–8, 9–12 points were separately considered as Negative, Low, Medium, and High. The results showed that the positive expression rate of B7-H3 protein in the pathological sections of 60 cases of osteosarcoma reached 73.3%, of which 8 cases (13.3%) had High grades and 18 cases (30%) had Medium grades, 18 cases (30%) of Low grades, and 16 cases (26.7%) of Negative expression (Fig. S3, Fig. S4 and Table 1). Representative immunohistochemical results showed that the B7-H3 protein was highly expressed on the cell membrane (Fig. 1A). Since we did not obtain the paracancerous tissue of osteosarcoma, we clearly observed that B7-H3 protein was highly expressed in osteosarcoma by referring to the expression level of B7-H3 in normal tumor tissue in other articles [24, 29]. In addition, we detected the expression of B7-H3 protein on four osteosarcoma cell lines (HOS, U-2 OS, SW1353, Saos-2) through flow cytometry, and the leukemia T cell line Jurkat was used as a negative control cell line. The results showed that all the four cell lines of osteosarcoma highly expressed the B7-H3 protein (Fig. 1B)

**Table 1:** B7-H3 positivity rate in OS samples

	**Total**	**High Positive**	**Medium Positive**	**Low Positive**	**Negative**	**Positive** **Rate(%)**
**OS**	**60**	**8**	**18**	**18**	**16**	**73.3**

### Construction of 3rd-generation CAR-T cells targeting B7-H3

**Fig. 2:**
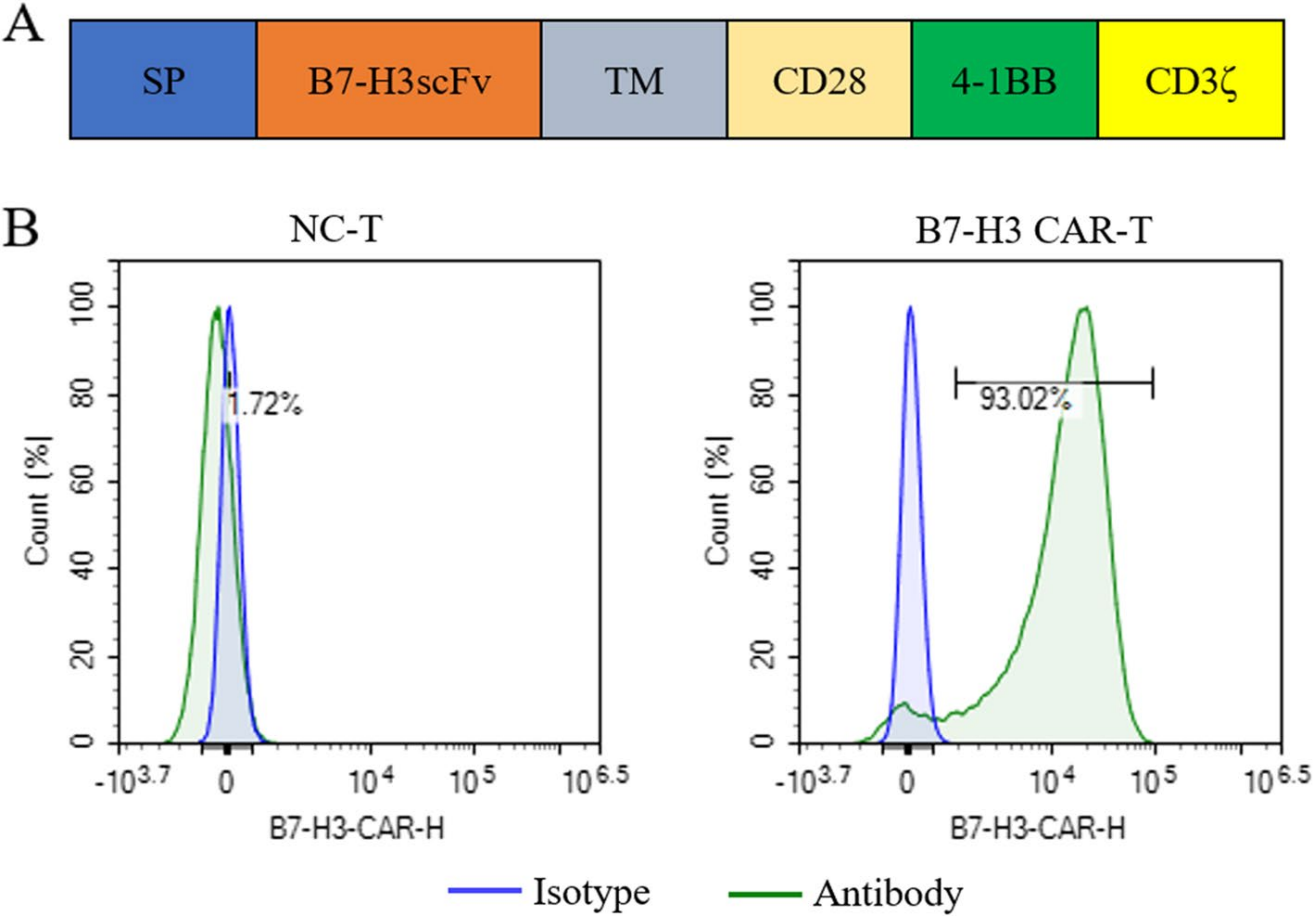
Construction of 3rd-generation CAR-T cells targeting B7-H3. **A** Schematic diagram of the B7-H3 CAR-T transgene. **B** Proportion of B7-H3 CAR infected primary human T cells determined by flow cytometry. C Histogram of the B7-H3-CAR-T rate of B7-H3 CAR infected cells and its control cells in three repeats. (mean ± SEM; ns not significant *P* > 0.05, *P < 0.05,**P < 0.01, ***P < 0.001)

Based on the results above (Fig. 1), B7-H3 has been proved to be an excellent potential target for osteosarcoma treatment. Thus, we proceeded to design the CAR structure targeting the B7-H3 target. The B7-H3 CAR contained a single-chain variable fragment of anti-B7-H3 antibody, a CD8 transmembrane domain, costimulatory domains including both CD28 and 4-1BB, and the T cell activating domain CD3ζ (Fig. 2A). The B7-H3 CAR lentivirus was produced by lentiviral packaging technology, and then the CAR lentivirus was used to infect T cells. The expression of the B7-H3 CAR on the T cells was detected through flow cytometry using the B7-H3 protein (Fig. 2B).

### Cell phenotype assay of B7-H3 CAR-T cell

**Fig. 3:**
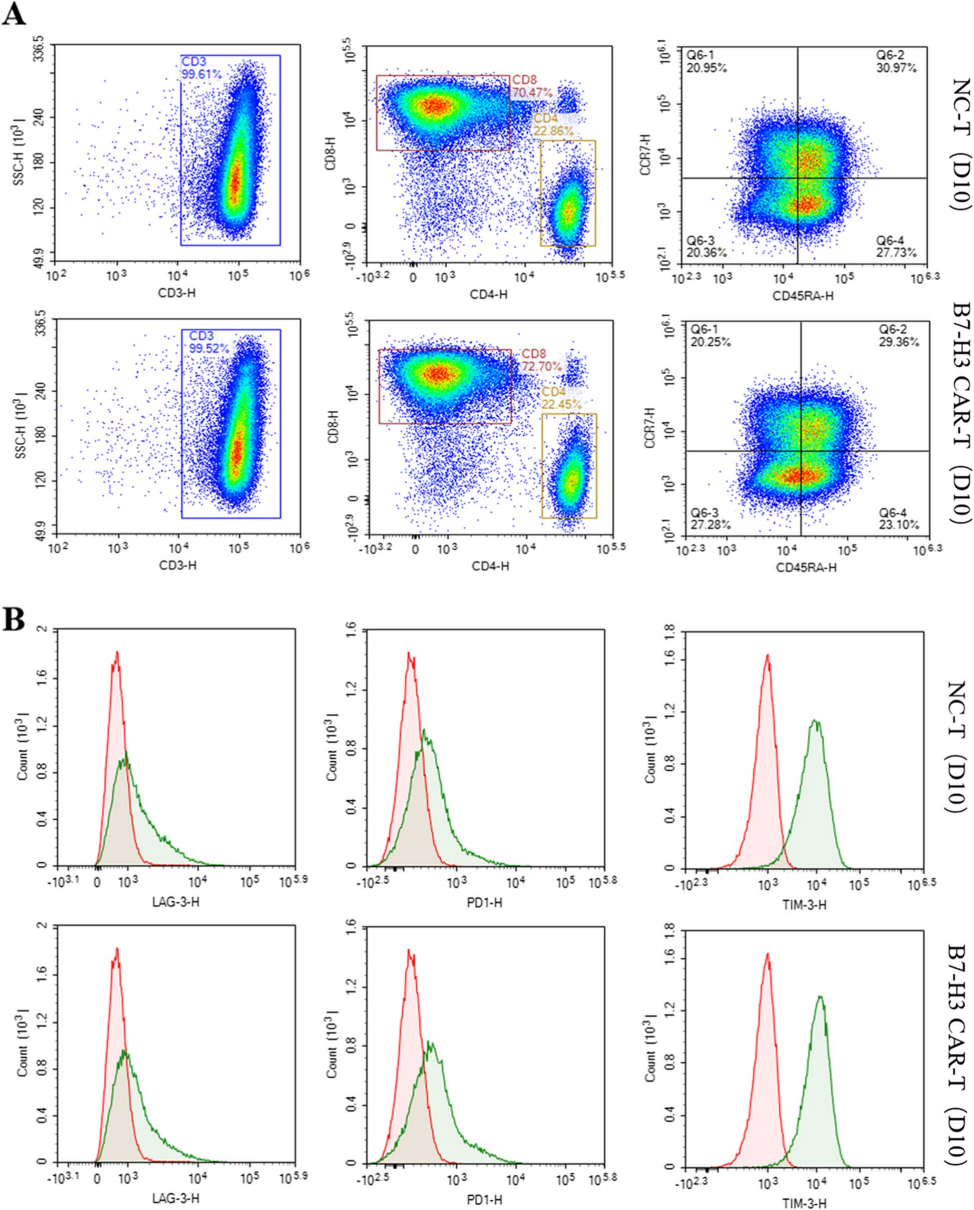
Cell phenotype of B7-H3 CAR-T cell. **A** Flow cytometry to detect CD3 + /CD4 + /CD8 + /CD45RA + /CCR7 + ratio in CAR-T cells and NC-T cells **B** Detection exhaustion biomarker of TIM3, PD1, LAG3 on CAR-T cells and NC-T cells by Flow cytometry.

When the B7-H3 CAR-T cells were cultured for 10 days, we analyzed the phenotype of T cells by flow cytometry. The results showed there not exist significant difference in phenotype between B7-H3 CAR-T cells and non-transduced T cells (NC-T). The purity of CD3 + T cells was very high, reaching more than 99%, the ratio of CD4/CD8 was also around 3:1, and the ratio of Tscm (CD45RA + CCR7 +) cells was also around 30%. (Fig. 3A) The comparative detection of the three immune checkpoint markers also showed no significant difference (Fig. 3B).Cell phenotyping experiments were performed on multiple samples, all of which showed similar results (results not shown). The results above showed that the B7-H3 CAR lentivirus infection had no obvious effect on the phenotype of T cells.

### Functional study of B7-H3 CAR-T cells *in vitro*

**Fig. 4:**
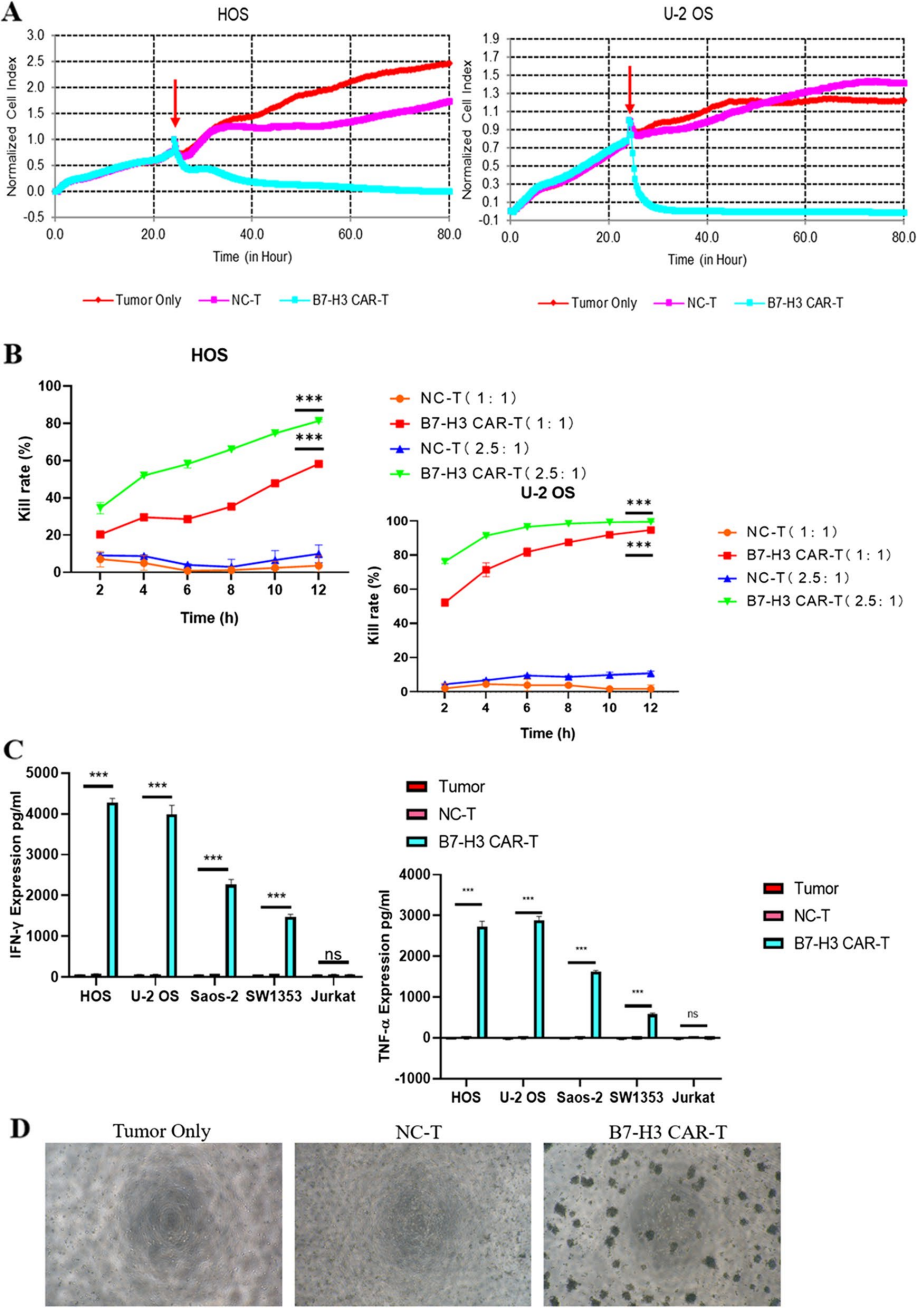
Functional Study of B7-H3 CAR-T cells *in vitro.* **A** The cytotoxicity of B7-H3 targeted CAR-T cells against solid tumor cell lines was analyzed by RTCA assay. The red arrow represents the time point of effector cell addition. **B** Comparison of killing rates under different E:T ratios on tumor cells. **C** Levels of IFNγ and TNFα released by B7-H3 CAR-T cells analyzed by ELISA after incubated with cells for 20 h. **D** Situation of effector cells and target cells (U-2 OS) displayed by optical microscopy. mean ± SEM; ns not significant *P* > 0.05, *P < 0.05, **P < 0.01, ***P < 0.001

When the B7-H3 CAR-T cells were cultured for 10 days, we respectively co-cultured B7-H3 CAR-T cells with 4 osteosarcoma cell lines (HOS, U-2 OS, SW1353, Saos-2) at an effector-to-target(E:T) ratio of 2.5:1, while the NC-T cell group and tumor only group were used as the control group in the experiment. During the co-incubation process, the RTCA instrument was used to observe the results in real time. The results showed that the B7-H3 CAR-T cells had an excellent killing effect on the four target-positive osteosarcoma cells, while the control group had no obvious change. (Fig. 4A, S1). After reducing E:T to 1:1, it also showed obvious tumor cell killing effect. In addition, the group using effector-to-target(E:T) ratio of 2.5:1 indicated higher tumor cell killing effect than that group using effector-to-target(E:T) ratio of 1:1, which showed that B7-H3 CAR-T cells had a manifest dose-dependent effect on tumor cell killing (Fig. 4B). To visually observe the killing effect of B7-H3 CAR-T cells against tumor cells, we repeated the tumor cell killing experiment using U-2 OS tumor cells in a 96-well cell culture plate, and the results clearly showed that the U-2 OS cells in the CAR-T cell group were significantly reduced, and the CAR-T cells clumped in the vicinity of the tumor cells (Fig. 4D). The results of the other three groups of osteosarcoma cells were shown in S2. Since the RTCA analysis system is more suitable for monitoring the experiments in which the target cells are adherent cells, the negative cell Jurkat is not involved in the above experiments. In experiments of detecting cytokine released by incubated cells, we used Jurkat cells as a target-negative group. Through detecting cytokine release in the supernatant after co-incubation for 18 h, we found that CAR-T cells in the B7-H3-positive osteosarcoma cell group significantly released cytokines including IFN-γ and TNF-α. However, both Jurkat cells and NC-T groups did not significantly release cytokines (Fig. 4C). The results above demonstrated that the effect of B7-H3 CAR-T cells on tumors was target-specific.

### Anti-tumor efficacy of B7-H3 CAR-T cells against PDX models *in vivo*

**Fig. 5:**
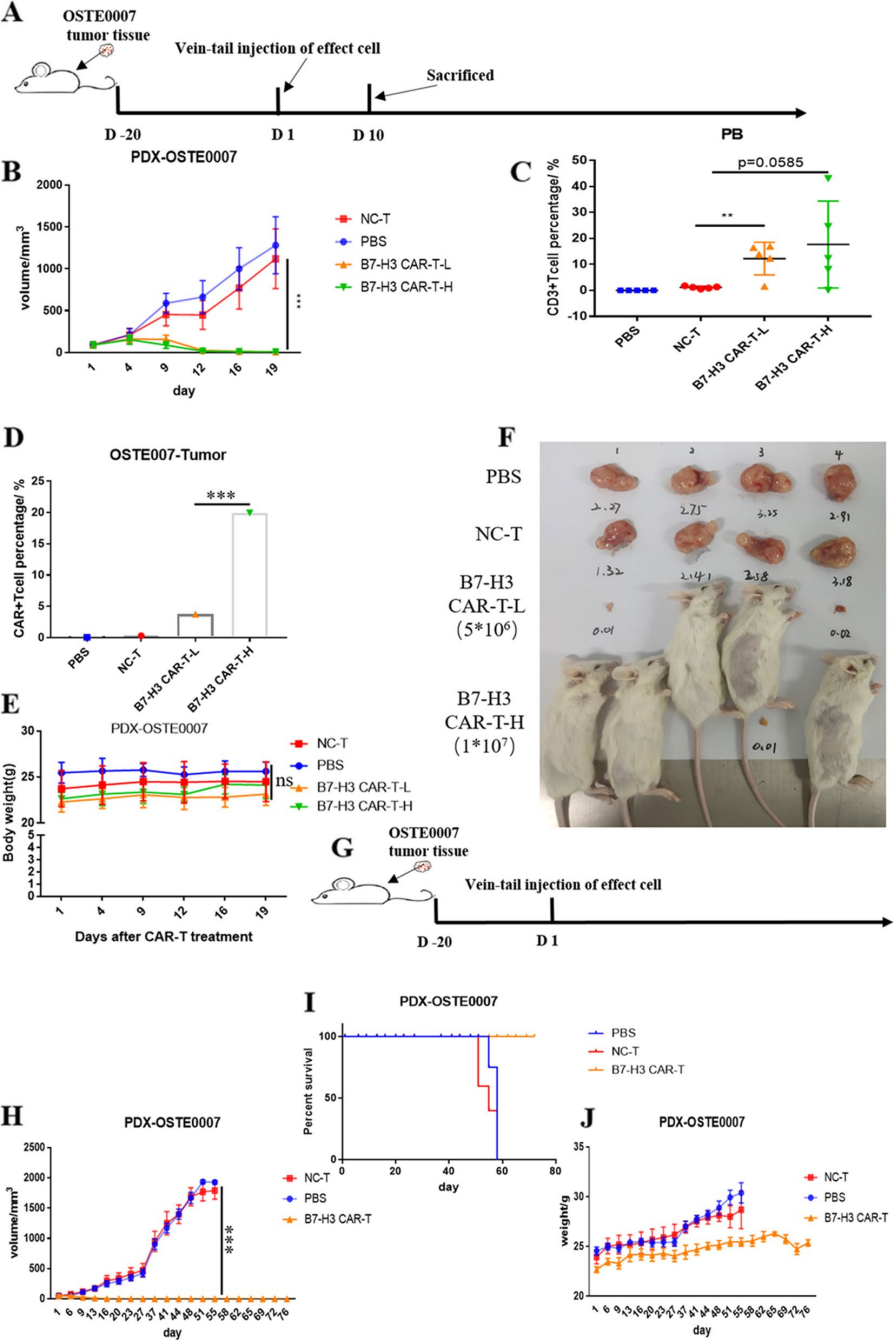
Anti-tumor efficacy of B7-H3 CAR-T cells against PDX models of osteosarcoma *in vivo*. **A** and **G** Schema of the experimental events and nodes. The tumor size **(B** and **H)** and mice weight (**F** and **J)** variation with B7-H3 CAR-T cells and control T cells injection among the Observed time. **C** Presence of CD3 + T cells in mouse peripheral blood after 10 days of injection. **D** Absence of CAR-T cells in mice tumors after 10 days of injection.** E** Size of tumor mass differed in B7-H3 CAR-T cells, NC T cells or PBS treated mice. **I** Kaplan–Meier curves for survival of the PDX-OSTE0007 models are shown. (mean ± SEM; ns not significant *P* > 0.05, *P < 0.05,**P < 0.01, ***P < 0.001)

For evaluating the effect of B7-H3 CAR-T cells on osteosarcoma *in vivo*, we constructed the osteosarcoma PDX models (named PDX-OSTE0007) using NCG mice. General protocol schema was illustrated in Fig. 5A. When the size of the transplanted tumor reached 50mm^3^, we randomly divided the mice into 4 groups (*n* = 5): PBS control group (named PBS in short), NC-T control group (named NC-T in short), B7-H3 CAR-T low-dose group (named B7-H3 CAR-T-L in short), B7- H3 CAR-T high-dose group (named B7-H3 CAR-T-H in short). The injected dose of CAR-T cells in the low-dose group was 5*10^6^CAR + cells per mouse, while the injected dose of CAR-T cells in the high-dose group was 1*10^7^CAR + cells per mouse. Significant tumor-suppressive effects were observed on the 7th day after injection. On the 10th day after injection, the blood was collected from all mice, then the proportion of CD3 + cells in peripheral blood was detected, and the results showed that the CAR-T cell group still had a large proportion, while the control NC T group had almost disappeared (Fig. 5C). In addition, one mouse per group was randomly selected for euthanasia and the tumor tissue was collected on the 10th day after injection. Then, the proportions of CAR-T cells in the tumor tissues were detected by flow cytometry. The results showed that the proportions of CAR-T cells in CAR-T injected groups were obvious (Fig. 5D). Tumors in the CAR-T cell groups disappeared or became very little since the 12th day after injection (Fig. 5B). We euthanized the mice on the 19th day after injection, and we observed that two mice in the low-dose CAR-T injected group were completely tumor-free, and three mice in the high-dose group were completely tumor-free (Fig. 5F). No difference in weight of mice was observed among the four groups above (Fig. 5E). Since the observation time of the first *in vivo* experiment was too short, we did the second *in vivo* experiment for a long-time observation (Fig. 5H). In the second in vivo experiment, when the size of the transplanted tumor reached 50mm^3^, we randomly divided the mice into 3 groups (*n* = 5): PBS control group (named PBS in short), NC-T control group (named NC-T in short), B7-H3 CAR-T group (named B7-H3 CAR-T in short, injected dose was 5*10^6^CAR + cells per mouse) (Fig. 5G). In the second *in vivo* experiment, B7-H3 CAR-T cells also had obvious tumor suppressive ability, which was consistent with the previous experiment. No tumor appeared in the mice in the CAR-T cell group within 80 days, and all the mice were healthy and alive (Fig. 5H, J). All the mice in the control group were euthanized due to oversized tumors around 50 days (Fig. 5I).

## Discussion

Selecting excellent targets is very important for CAR-T cell therapy. The most ideal targets (named tumor-specific antigen (TSA)) are specifically expressed on the tumors but not expressed on the normal tissues. Unfortunately, the number of TSA is few, and TSA only exists in a few people, both of which severely limits its application in CAR-T cell therapy. Therefore, the targets used in current CAR-T cell therapy are tumor-associated antigens (TAAs), which are expressed very low on normal tissues but highly expressed on tumors. CAR-T cell products targeting CD19 or BCMA were already on the market, and both CD19 and BCMA were TAA targets. By performing IHC on clinical tissue samples of osteosarcoma, we found that the positive rate of B7-H3 target in osteosarcoma tissue was as high as 73.3% (Fig. S3). Other reports also mentioned that B7-H3 target was highly expressed in various tumor tissues including glioma, anaplastic meningioma, chordomas, panic ductal adenocarcinoma (PDAC) and ovarian cancer [26, 29, 30].

Osteosarcoma is one of the foremost common dangerous bone tumors in children. Until now, the main treatments for osteosarcoma are still urgery, radiotherapy and chemotherapy, but the effect of the above treatments are limited [3, 4]. Compared with other cancers such as colorectal cancer and gastric cancer, there are very few projects developed for the target of osteosarcoma. According to the *clinicaltrials.gov* queried on the clinical trial website, there are very few clinical trials for osteosarcoma. Therefore, new and effective therapies for osteosarcoma treatment are urgently needed.

We designed *in vitro* killing experiments on osteosarcoma cell lines by constructing third-generation CAR-T cells targeting B7-H3 antigen. The results in Fig. 4 showed that B7-H3 targeted CAR-T cells could specifically recognize target-positive tumor cells and released cytokines to cause cell killing. In *in vivo* experiments, we firstly constructed a mouse PDX model derived from the tumor tissue of patients with osteosarcoma which could simulate the actual tumor tissue environment. In this model, B7-H3 targeted CAR-T cells showed excellent anti-tumor effects, and both the high-dose group and the low-dose group showed efficient tumor inhibition. In addition, even in the long-time experiment in the low-dose group, the tumor did not recur (Fig. 5H).

The effect of CAR-T cell therapy is significant in treating hematologic malignancies, and several CAR-T cell products for hematologic malignancy treatment that have been on market globally. However, in the treatment of solid tumors, CAR-T cell therapy is faced with enormous difficulties. The solid tumors have more hidden presence or more complicated tumor microenvironments compared to the hematologic malignancies, which may limit the use of CAR-T cell therapy for treating solid tumor [31, 32]. Some studies showed that addition of cytokines and chemokines could enhance the anti-tumor effect of CAR-T cells in mice [33, 34].Some other studies also demonstrated that the effects of CAR-T cells against solid tumors could be enhanced by combined with PD-1 antibodies, PD-L1 antibodies [35, 36]. The previous studies above offered us new ideas for improving our product in this study.

Based on the results in our study, B7-H3 was proved to be a general target of solid tumor treatment, and the B7-H3 targeted CAR-T cell therapy we developed showed excellent effect for treating osteosarcoma. We will design and conduct the clinical trail for our product and develop more excellent CAR-T cell therapies for osteosarcoma and other solid tumor treatment in the future.

## Conclusion

The B7-H3 targeted CAR-T cells we constructed showed the strong B7-H3-specific tumor cell killing ability *in vitro* and significant tumor-suppression effect in the PDX model *in vivo*, which demonstrated its enormous potency to be used for treating osteosarcoma in the clinical trials in the future.

## Supplementary Information

**Additional file 1:** **Supplemental Figure 1.** The cytotoxicity of B7-H3CAR-T cells against solid tumor cell lines was analyzed by RTCA assay. The redarrow represents the time point of effector cell addition.

**Additional file 2:** **Supplemental Figure 2.** Situation of affectorcells and target cells (HOS, Saos-2, SW1353) displayed by optical microscopy.

**Additional file 3:** **Supplemental Figure 3.** Negative and lowgrades of B7-H3 expression in the pathological sections of 60 cases ofosteosarcoma.

**Additional file 4:** **Supplemental Figure 4.** Medium and highgrades of B7-H3 expression in the pathological sections of 60 cases ofosteosarcoma.

## Data Availability

The datasets supporting the conclusions of this study are included in the article and supplemental data. For detailed and original data, please contact Mr. Qian Zhang at zq@yihaobio.com, or corresponding author.

## Abbreviations

OS: Osteosarcoma
CAR-T: Chimeric antigen receptor T
RTCA: Real Time Cell Analysis
PDX: Patient-derived xenografts
IFN-γ: Interferon-γ
TNF-α: Tumour necrosis factor-α
TAA: Tumor-associated antigen
