# Supplementary figures and images for: B7-H3 targeted CAR-T cells show highly efficient anti-tumor function against osteosarcoma both in vitro and in vivo
Source: BMC Cancer. 2022 Nov 2;22:1124. doi: 10.1186/s12885-022-10229-8 (PMC9628043; doi:10.1186/s12885-022-10229-8)

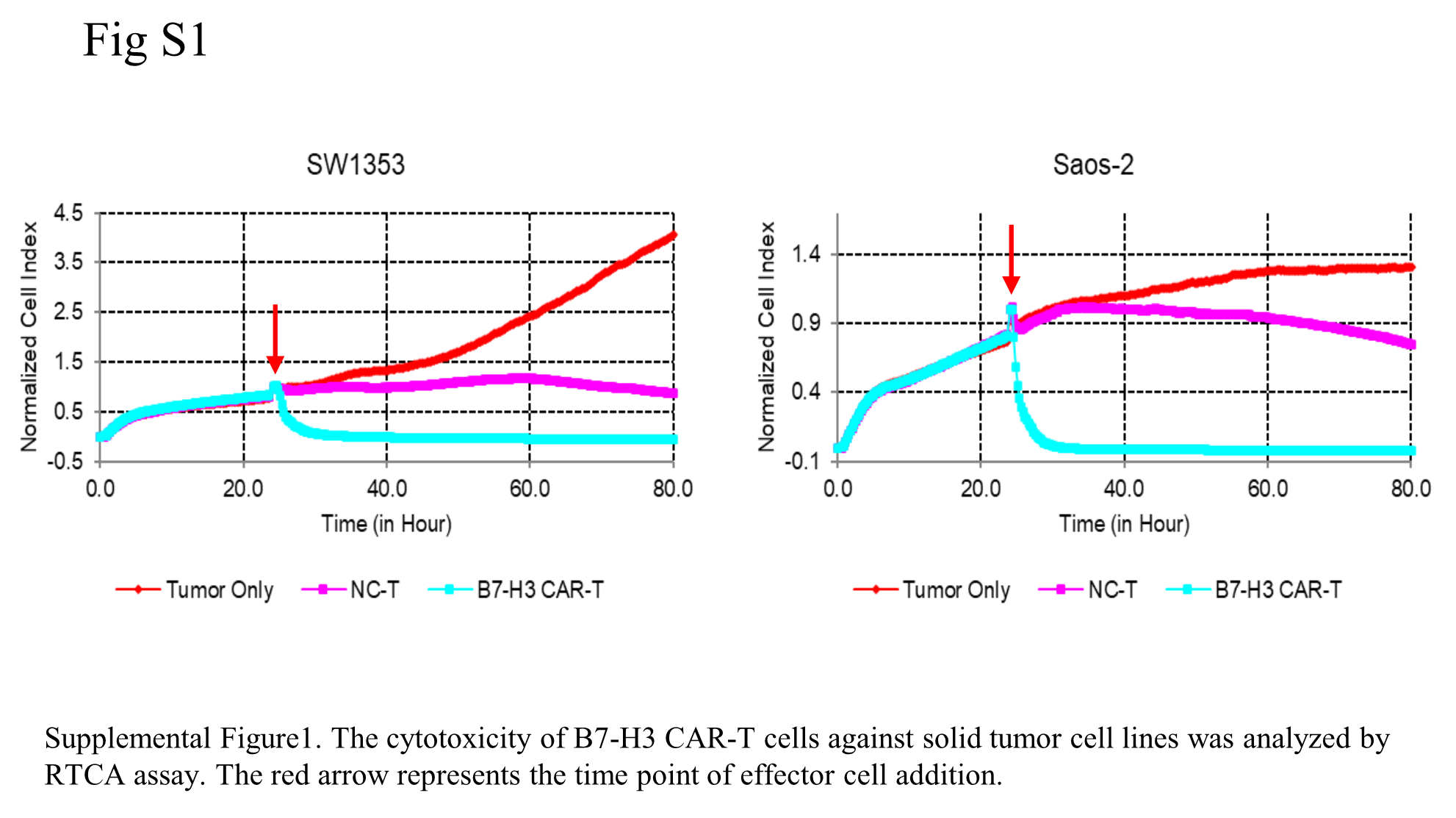

Supplement: Supplementary file 1 — Additional file 1: Supplemental Figure 1. The cytotoxicity of B7-H3CAR-T cells against solid tumor cell lines was analyzed by RTCA assay. The redarrow represents the time point of effector cell addition. [file 12885_2022_10229_MOESM1_ESM.tiff]

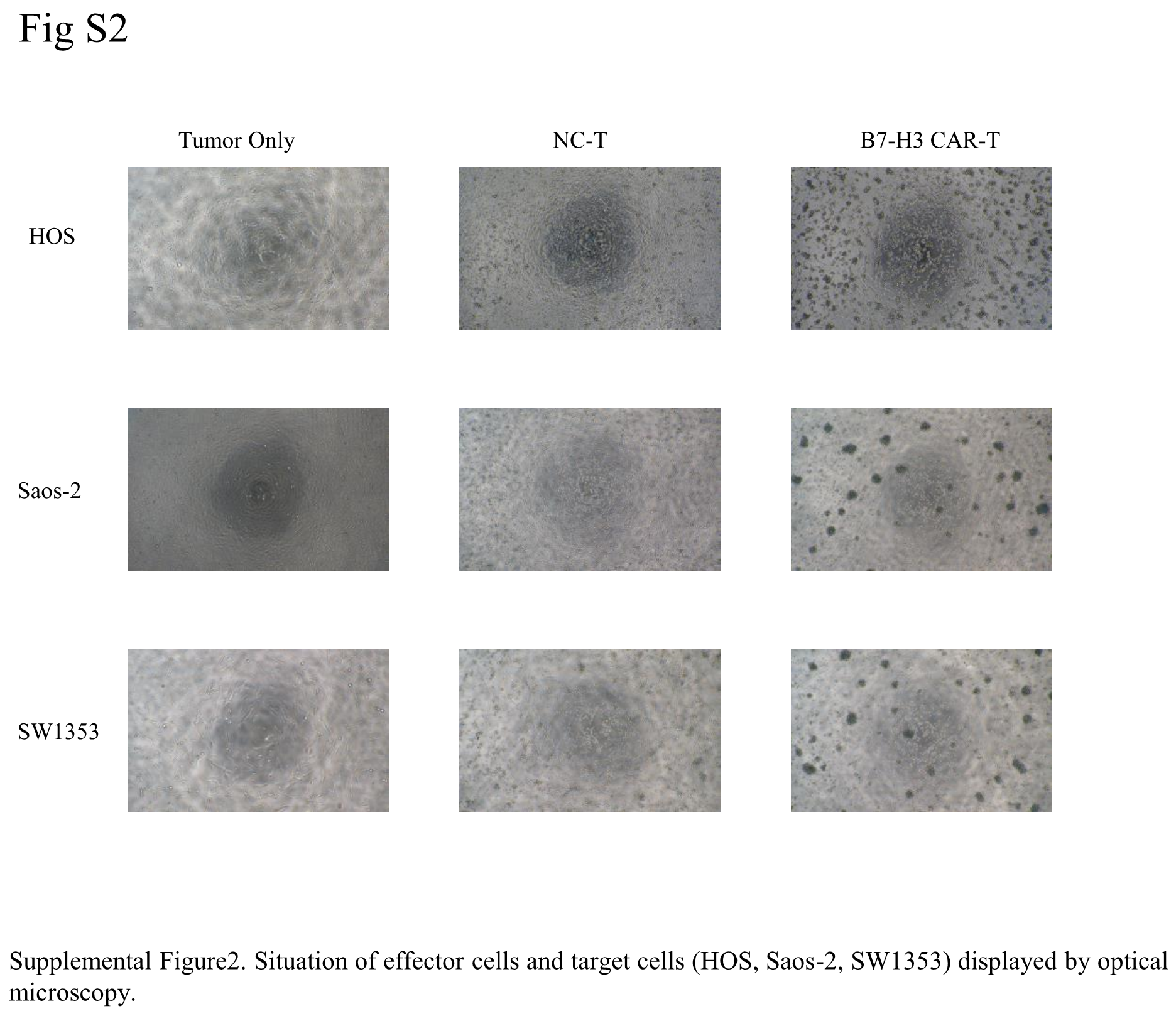

Supplement: Supplementary file 2 — Additional file 2: Supplemental Figure 2. Situation of affectorcells and target cells (HOS, Saos-2, SW1353) displayed by optical microscopy. [file 12885_2022_10229_MOESM2_ESM.tiff]

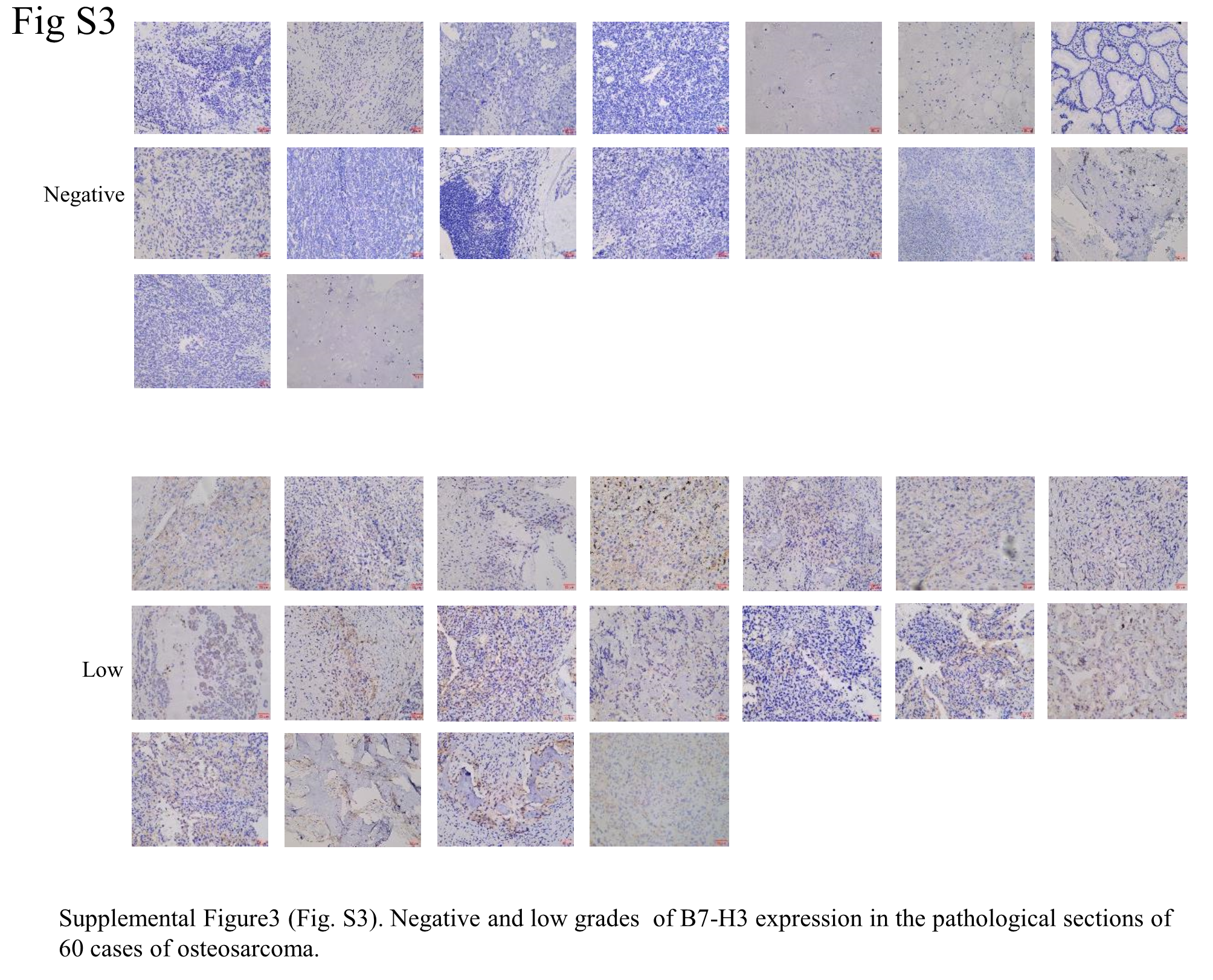

Supplement: Supplementary file 3 — Additional file 3: Supplemental Figure 3. Negative and lowgrades of B7-H3 expression in the pathological sections of 60 cases ofosteosarcoma. [file 12885_2022_10229_MOESM3_ESM.tiff]

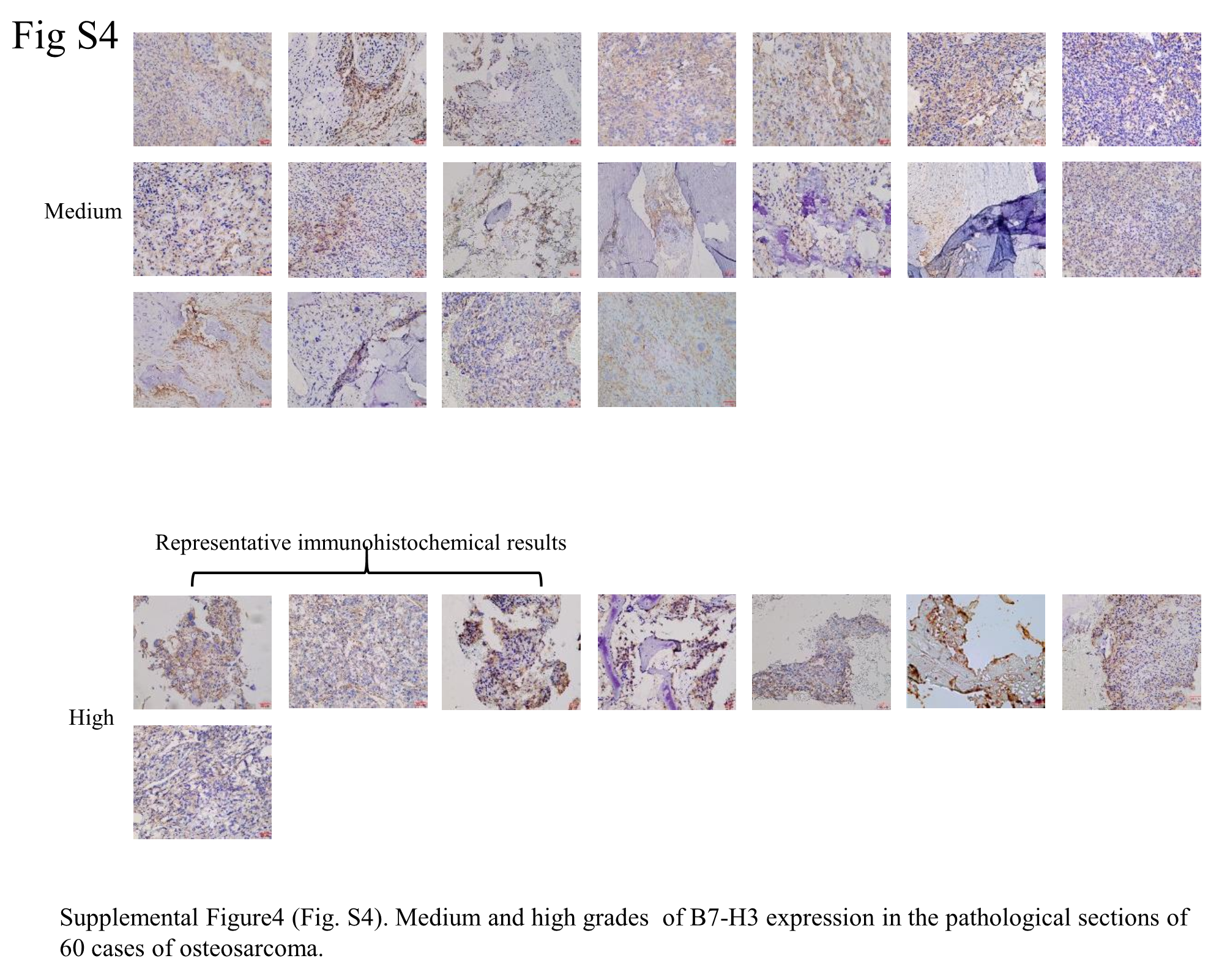

Supplement: Supplementary file 4 — Additional file 4: Supplemental Figure 4. Medium and highgrades of B7-H3 expression in the pathological sections of 60 cases ofosteosarcoma. [file 12885_2022_10229_MOESM4_ESM.tiff]
